# *Kytococcus schroeteri* Bacteremia in a Patient with Hairy Cell Leukemia: A Case Report and Review of the Literature

**DOI:** 10.1155/2015/217307

**Published:** 2015-05-07

**Authors:** Akshay Amaraneni, Devin Malik, Sakshi Jasra, Sreenivasa R. Chandana, Deepak Garg

**Affiliations:** ^1^Department of Internal Medicine, Western Michigan University Homer Stryker M.D. School of Medicine, 1000 Oakland Drive, Kalamazoo, MI 49008, USA; ^2^Department of Internal Medicine, University at Buffalo School of Medicine, Buffalo, NY 14260, USA; ^3^Department of Hematology and Oncology, West Michigan Cancer Center, 200 N. Park Street, Kalamazoo, MI 49007, USA

## Abstract

The *Kytococcus* genus formerly belonged to *Micrococcus*. The first report of a *Kytococcus schroeteri* infection was in 2002 in a patient diagnosed with endocarditis. We report a case of central line associated *Kytococcus schroeteri* bacteremia in a patient with underlying Hairy Cell Leukemia. *Kytococcus schroeteri* is an emerging infection in the neutropenic population and in patients with implanted artificial tissue. It is thought to be a commensal bacterium of the skin; however, attempts to culture the bacteria remain unsuccessful. There have been a total of 5 cases (including ours) of *K. schroeteri* bacteremia in patients with hematologic malignancies and neutropenia and only 18 documented cases in any population. Four of the cases of bacteria in neutropenic patients have been fatal, but early detection and treatment could make a difference in clinical outcomes.

## 1. Introduction

*Kytococcus schroeteri* was first identified in 2002 in a patient with endocarditis and bacteremia [1]. The* Kytococcus* genus formerly belonged to* Micrococcus* and was recently changed due to chemotaxonomic and phylogenetic grouping [1]. To date, there have been few situations where* Kytococcus* species have been pathogenic [2–6]. Patients with hematologic malignancies are immunocompromised and are prone to opportunistic infections from nonharmful bacteria. Here, we report a rare case of* Kytococcus schroeteri* sepsis in a patient with Hairy Cell Leukemia.

## 2. Case Report

A fifty-year-old Caucasian woman presented with fever, malaise, and pancytopenia. Her surgical history was significant for a splenectomy following a motor vehicle accident several years ago, and her vaccination status was up to date. Bone marrow biopsy revealed a diagnosis of Hairy Cell Leukemia. Immunophenotyping by flow cytometry was positive for CD11c, CD25, CD103, CD19, and CD20. The patient was started on Cladribine (2-chlorodeoxyadenosine), a purine analog, 0.14 mg/kg/day, for a five-day course. Chemotherapy was administered through a central venous catheter. While receiving Cladribine, the patient was noted to have persistent fevers with stable hemodynamics. After carefully excluding infectious etiologies of her persistent fever intravenous Cefepime was changed to oral ciprofloxacin and prophylactic fluconazole. Upon discharge, blood and sputum cultures were negative for bacteria.

One week later, she presented with persistent fevers and dyspnea. She was intubated and transferred to the Intensive Care Unit. Chest X-ray showed a left lower lobe infiltrate. She was started on vancomycin, piperacillin/tazobactam, and levofloxacin for healthcare associated pneumonia in the setting of neutropenic fever. Liposomal amphotericin-B was also added for broad-spectrum antifungal coverage as she was at high risk for developing fungal infection.

Bronchoscopy, performed at bedside, revealed diffuse petechial lesions in the airway. Bronchoalveolar lavage (BAL) culture grew greater than 10,000 colonies of a gram-positive organism that grew in clusters but was neither* Staphylococcus aureus* nor coagulase negative* Staphylococcus* when tested by fluorescent* in situ* hybridization using peptide nucleic acid probes (PNA FISH). The patient's blood cultures were also positive for the same nonstaphylococcal gram-positive bacteria in two separate aerobic culture tubes. Because the bacterium was unidentified, the patient remained on vancomycin. However, despite aggressive treatment, the patient deteriorated and expired four days after hospitalization. Our facility could not identify the bacteria and the sample was sent to the Department of Community Health of Michigan. The isolate was identified via sequencing of the 16S rDNA segment as* Kytococcus schroeteri*. Because the bacterium was detected posthumously, no susceptibility testing was performed by our facility and no samples were available to perform testing after the bacterium was identified.

## 3. Discussion

*K. schroeteri* is a recently discovered member of the genus* Kytococcus* that is thought to be a commensal organism of the human skin [1, 7]. To date, however, attempts to culture* K. schroeteri* from patients have been unsuccessful [6, 7]. It is true habitat remains undiscovered. We are not sure whether the* K. schroeteri* pneumonia in our patient was a primary infection or a secondary manifestation of central line associated* K. schroeteri* bacteremia.

There have been 5 documented cases of* K. schroeteri* infection in patients with hematologic malignancies. In the other 4 patients reported in the literature with hematologic malignancies and* K. schroeteri* infection, the underlying malignancy was AML. All 5 patients had pulmonary infiltration, 4 patients had bacteremia, and 1 patient had skin manifestations. Four out of 5 reported patients died. All of the patients developed their infection after induction chemotherapy. We believe the central venous catheter placement in our patient had a role in the development of this infection. There are several other cases of* K. schroeteri* infection that prove the bacterium has a propensity to favor synthetic material as a site of infection [1, 5, 6]. Our patient likely developed a central line associated blood stream infection and eventual seeding of the pulmonary tissue leading to pneumonia. In addition, there is one case of community-acquired pneumonia in a patient who was immunosuppressed secondary to daily administration of 20 mg of Prednisone. This patient developed bacteremia as well died from septic shock [8]. Given the presence of these 6 cases of pneumonia with bacteremia does not make it unreasonable to hypothesize that* K. schroeteri* has some adhesive properties that allow it to stick to the surface epithelium of the respiratory tract and cause patients to develop pneumonia in the appropriate clinical setting. In addition to the 6 cases of bacteremia and pneumonia, we have reviewed the literature and found 12 cases of implantable tissue related infections caused by* K. schroeteri*. These cases are detailed in Table 1.

*K. schroeteri* is an underrecognized and underreported organism. Hodiamont et al. reported on two cases of pneumonia and bacteremia caused by* K. schroeteri* [4]. In their research they came across six cases of pneumonia and bacteremia that were previously identified as* Micrococcus* sp. related. The discrepancies in those cases came from the fact that while the bacteria were identified as* Micrococcus* sp., they were resistant to penicillins. One of the important therapeutic properties of* Micrococcus* species is that they are inherently susceptible to penicillin and oxacillin [1]; however,* Kytococcus* species are not [1–6].

In conclusion, there are many factors involved in this case. The patient's underlying hematologic malignancy leading to prolonged neutropenia is likely the most important factor. In addition, the treatment with chemotherapy, history of splenectomy, and placement of a central venous catheter also likely contributed to the severity of infection in our patient. However, despite appropriate antibiotic therapy, the patient did not recover and eventually expired due to the infection. One of the unfortunate limitations in our case is the lack of sensitivity testing. We are not sure this would have changed the outcome in our patient due to the degree of overwhelming septic shock and multiorgan dysfunction; however, sensitivity testing may have impacted our treatment choices in this patient. Other cases outlined the use of different antibiotics including linezolid [3], doxycycline [5], amoxicillin [9], ofloxacin [10], and daptomycin [11]. However, vancomycin remains a common initial option due to the gram-positive nature of the organism.

Further studies should concentrate on the reason why prosthetic infections due to* K. schroeteri* are far less virulent compared to pneumonia and bacteremia. So far all 12 documented prosthetic tissue infections have resulted in no deaths, while 5 out 6 patients with pneumonia and bacteremia have died. The immunocompromised status of the 5 patients that died is likely the most important reason; however, this is difficult to confirm based on the dearth of case reports. At this point, it is not clear whether the immense immunocompromised status due to prolonged neutropenia played a role in a worse outcome or if it is due to unrecognized virulence factors of this organism.

## Figures and Tables

**Table 1:** A review of the cases of *Kytococcus schroeteri* in patients with hematologic malignancies.

Case	Age/sex	Condition	Underlying malignancy	Implanted device	Antibiotics used	Outcome
Nagler et al., 2011 [2]	68 M	Papular skin rash, pneumonia, and bacteremia	Acute myeloid leukemia	Absent	Vancomycin	Deceased

Blennow et al., 2011 [3]	43 F	Pneumonia and bacteremia	Acute myeloid leukemia	Absent	Vancomycin/piperacillin/tazobactam, vancomycin/meropenem, linezolid, discharged on TMP/SMX	Discharged

Hodiamont et al., 2010 [4]	40 M	Pneumonia/bacteremia	Acute myeloid leukemia	Absent	Vancomycin, rifampin, gentamicin	Deceased

Hodiamont et al., 2010 [4]	52 M	Pneumonia/bacteremia	Acute myeloid leukemia	Central venous catheter	Vancomycin, ceftazidime	Deceased

Chan et al., 2012 [5]	45 M	Artificial tissue infection	None	Silicone tendon graft	Surgery, oral doxycycline (6 weeks)	Discharged

Le Brun et al., 2005 [6]	73 M	Endocarditis	None	Bioprosthetic Aortic valve	Surgery followed by teicoplanin (6 weeks), rifampin and gentamicin (both 3 weeks)	Discharged

Mohammedi et al., 2005 [8]	71 F	Pneumonia/bacteremia	Asthma on prednisone	None	Ceftriaxone, ofloxacin	Deceased

Becker et al., 2003 [12]	34 F	Endocarditis	None	Mechanical Aortic valve	Vancomycin, rifampin, and gentamicin	Discharged

Mnif et al., 2006 [13]	49 F	Endocarditis	None	Artificial Mitral valve	Vancomycin, gentamicin followed by pristinamycin, and vancomycin	Discharged

Aepinus et al., 2007 [14]	38 F	Endocarditis	None	Mechanical Aortic valve	Vancomycin, rifampin (6 weeks), gentamicin (2 weeks), followed by levofloxacin and rifampin (2 months)	Discharged

Renvoise et al., 2007 [9]	70 M	Endocarditis	None	Bioprosthetic Aortic valve	Amoxicillin (6 weeks), gentamicin (2 weeks) followed by surgery	Discharged

Jourdain et al., 2009 [15]	13 month M	Artificial tissue infection	Hydrocephalus	Ventriculoperitoneal shunt	Vancomycin and rifampin (27 days) with surgery 4 days into treatment	Discharged

Poyet et al., 2010 [16]	72 M	Endocarditis	None	Mechanical Aortic valve	Vancomycin and rifampin (6 weeks), gentamicin (2 weeks)	Discharge

Yousri et al., 2010 [17]	64 M	Endocarditis and root abscess	None	Mechanical Aortic valve	Surgery followed by 6 weeks of antibiotics.	Discharged

Jacquier et al., 2010 [10]	50 F	Artificial discitis	Type 2 diabetes mellitus	Prosthetic L3-L4 disc.	Ofloxacin and rifampin (6 weeks)	Discharged

Liu et al., 2012 [11]	53 M	Endocarditis	None	Bioprosthetic Aortic valve	Daptomycin (8 weeks)	Discharged

Schaumburg et al., 2013 [18]	3 yrs 9 months F	Artificial tissue infection	Ganglioma treated with surgical resection	Ventriculoperitoneal shunt	Surgery followed by cefuroxime (23 days), gentamicin (22 days)	Discharged

Amaraneni et al., 2015 (Our case)	51 F	Pneumonia/bacteremia	Hairy Cell Leukemia	Central Venous Cather	Vancomycin, piperacillin/tazobactam	Deceased
